# (*R*)-(−)-3-Hydroxy­quinuclidinium chloride

**DOI:** 10.1107/S1600536808009185

**Published:** 2008-04-16

**Authors:** Miłosz Siczek, Tadeusz Lis

**Affiliations:** a Faculty of Chemistry, University of Wrocław, 14 Joliot-Curie St, 50-383 Wrocław, Poland

## Abstract

The quinuclidinium cation of the title compound, C_7_H_14_NO^+^·Cl^−^, shows a twist along the C—N pseudo-threefold axis, with N—C—C—C torsion angles of −16.0 (1), −16.9 (1) and −15.6 (1)°. The crystal structure is stabilized by N—H⋯Cl and O—H⋯Cl hydrogen bonds, forming infinite chains along the *a* and *b* axes.

## Related literature

For related literature see: Carroll *et al.* (1991 ▶); Erman *et al.* (1994 ▶); Frackenpohl & Hoffmann (2000 ▶); Bosak *et al.* (2005 ▶); Lis & Jeżowska-Trzebiatowska (1976 ▶); Lis *et al.* (1975 ▶); Morrow (1962 ▶); Noddack & Noddack (1933 ▶); Sterling *et al.* (1988 ▶).
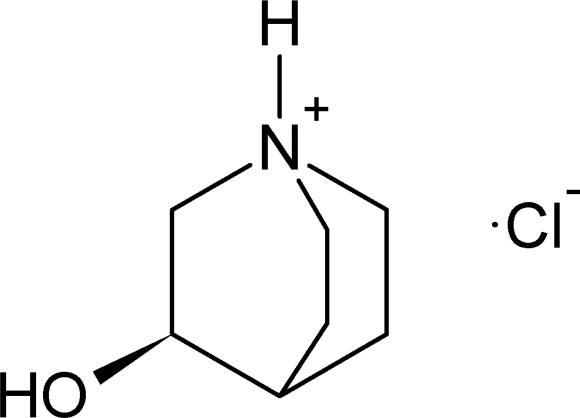


## Experimental

### 

#### Crystal data

C_7_H_14_NO^+^·Cl^−^
*M* *_r_* = 163.64
Tetragonal, 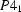

*a* = 6.655 (3) Å
*c* = 18.145 (9) Å
*V* = 803.6 (6) Å^3^
*Z* = 4
Mo *K*α radiation
μ = 0.41 mm^−1^
*T* = 100 (2) K
0.50 × 0.34 × 0.08 mm

#### Data collection

Kuma KM-4 CCD κ-geometry diffractometer
Absorption correction: analytical (*CrysAlis RED*; Oxford Diffraction, 2007 ▶) *T*_min_ = 0.86, *T*_max_ = 0.97
11241 measured reflections
3310 independent reflections
3128 reflections with *I* > 2σ(*I*)
*R*_int_ = 0.022

#### Refinement

*R*[*F* ^2^ > 2σ(*F* ^2^)] = 0.026
*wR*(*F* ^2^) = 0.063
*S* = 1.00
3310 reflections
92 parameters
1 restraint
H-atom parameters constrained
Δρ_max_ = 0.36 e Å^−3^
Δρ_min_ = −0.20 e Å^−3^
Absolute structure: Flack (1983 ▶), 1361 Friedel pairs
Flack parameter: −0.01 (3)

### 

Data collection: *CrysAlis CCD* (Oxford Diffraction, 2007 ▶); cell refinement: *CrysAlis RED* (Oxford Diffraction, 2007 ▶); data reduction: *CrysAlis RED*; program(s) used to solve structure: *SHELXS97* (Sheldrick, 2008 ▶); program(s) used to refine structure: *SHELXL97* (Sheldrick, 2008 ▶); molecular graphics: *XP* in *SHELXTL* (Sheldrick, 2008 ▶); software used to prepare material for publication: *SHELXL97*.

## Supplementary Material

Crystal structure: contains datablocks I, global. DOI: 10.1107/S1600536808009185/pk2091sup1.cif

Structure factors: contains datablocks I. DOI: 10.1107/S1600536808009185/pk2091Isup2.hkl

Additional supplementary materials:  crystallographic information; 3D view; checkCIF report

## Figures and Tables

**Table 1:** Hydrogen-bond geometry (Å, °)

*D*—H⋯*A*	*D*—H	H⋯*A*	*D*⋯*A*	*D*—H⋯*A*
N1—H1⋯Cl	0.93	2.14	3.060 (2)	171
O1—H11⋯Cl^i^	0.84	2.24	3.079 (2)	173

Symmetry code: (i) 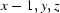.

**Fig. 1.**
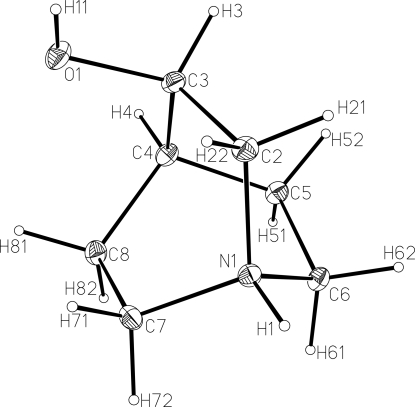
A view of (R)-(-)-3-hydroxyquinuclidinium cation with atom labelling scheme. The thermal displacement ellipsoids are drawn at 30% probability level.

**Fig. 2.**
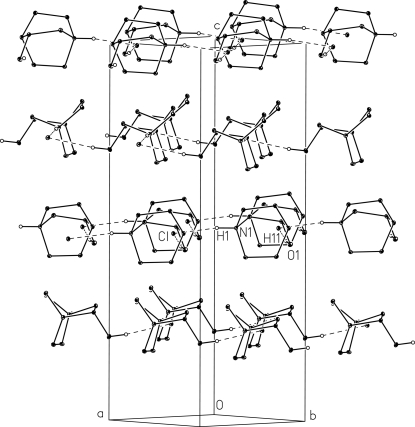
A view of molecular packing showing chains formed along a and b directions. The hydrogen bonds are shown as dashed lines. The H atoms not involved in any interaction are omitted for clarity.

**Fig. 3.**
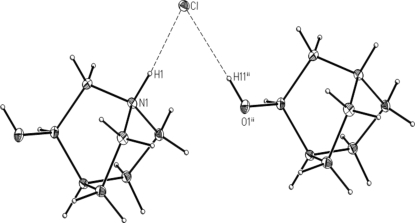
A view of (R)-(-)-3-hydroxyquinuclidinium cations and chloride anion forming hydrogen bonds. The thermal displacement ellipsoids are drawn at 30% probability level. Symmetry code: [ii] x + 1,y,z.

## supplementary crystallographic information

### Comment

Since the first synthesis of potassium µ-oxo-bis[pentachlororhenate(IV)] (Noddack & Noddack, 1933) numerous efforts have been undertaken to quantitatively describe its structure. To the present day only one structure of [Re_2_OCl_10_]^4-^ with potassium cations and one of its oxidized form, [Re_2_OCl_10_]^3-^, with caesium cations have been successfully determined by X-Ray crystallography (Morrow, 1962; Lis & Jeżowska-Trzebiatowska, 1976; Lis *et al.*, 1975;). Our structural studies on [Re_2_OCl_10_]^4-^ and [Re_2_OCl_10_]^3-^ have shown that the appropriate choice of cation is crucial to obtain good structural parameters for the anion unit. The most suitable properties of the cation are low symmetry, chirality and the ability to form hydrogen bonds. All these requirements are fulfilled by (*R*)-(–)-3-hydroxyquinuclidinium cation. Quinuclidinium derivatives have been of interest due to their biological activity, especially as a acetylcholinesterase inhibitor (Bosak *et al.*, 2005). It was also proven that quinuclidinium salts protected rats against the toxicity of soman and tabun (Sterling *et al.*, 1988).  Aside from the present study, the only other known structure of (*R*)-(–)-3-hydroxyquinuclidinium was with (*R*,*R*)-tartrate anion (Erman *et al.*, 1994).

The asymmetric unit of the crystal (Fig. 1) consists of a (*R*)-(–)-3-hydroxyquinuclidinium cation and a chloride anion. The quinuclidine moiety has almost exact threefold symmetry about N1–C4, and the two subunits (N1, C2, C6, C7 and C4, C3, C5, C8) are twisted about this axis. The deformation of quinuclidinium cation is reflected in the values of the N1—C2—C3—C4, N1—C6—C5—C4, N1—C7—C8—C4 torsion angles, which are -16.0 (1)° -16.9 (1)° -15.6 (1)°, respectively. Similar rotation has also been observed, but with slightly smaller angles, in 3-hydroxyquinuclidinium tartrate (Erman *et al.*, 1994). The bond lengths of the cation are all normal and are in good agreement with quinuclidinium derivatives (Carroll *et al.*, 1991; Erman *et al.*, 1994; Frackenpohl & Hoffmann, 2000). The anion is surrounded by six symmetry-related cations that act as hydrogen bond acceptors for O—H and N—H groups. The hydrogen bonds link cations and anions into infinite chains running in the *a* and *b* axis directions (Figs. 2,3).

### Experimental

The title compound was obtained from a commercial source (Aldrich) and dissolved in hot methanol.  Colourless crystals grew from the solution after a few hours.

### Refinement

The H atoms firstly were all located in difference maps, then set in calculated positions and refined as riding atoms [C—H = 0.99–1.00 Å, O—H = 0.84 Å and *U*_iso_(H) = 1.2Ueq(C) or 1.5Ueq(O)].

### Crystal data

**Table:**

C_7_H_14_NO^+^·Cl^–^	*Z* = 4
*M**_r_* = 163.64	*F*_000_ = 352
Tetragonal, *P*4_1_	*D*_x_ = 1.353 Mg m^−^^3^
Hall symbol: P 4w	Mo *K*α radiation λ = 0.71073 Å
*a* = 6.655 (3) Å	Cell parameters from 11099 reflections
*b* = 6.655 (3) Å	θ = 3.3–36.6º
*c* = 18.145 (9) Å	µ = 0.41 mm^−^^1^
α = 90º	*T* = 100 (2) K
β = 90º	Plate, colorless
γ = 90º	0.50 × 0.34 × 0.08 mm
*V* = 803.6 (6) Å^3^	

### Data collection

**Table:**

Kuma KM-4-CCD κ-geometry diffractometer	3310 independent reflections
Radiation source: medium-focus sealed tube	3128 reflections with *I* > 2σ(*I*)
Monochromator: graphite	*R*_int_ = 0.022
*T* = 100(2) K	θ_max_ = 36.7º
ω scans	θ_min_ = 3.3º
Absorption correction: analytical(CrysAlis RED; Oxford Diffraction, 2007)	*h* = −11→8
*T*_min_ = 0.86, *T*_max_ = 0.97	*k* = −8→11
11241 measured reflections	*l* = −23→30

### Refinement

**Table:**

Refinement on *F*^2^	Hydrogen site location: inferred from neighbouring sites
Least-squares matrix: full	H-atom parameters constrained
*R*[*F*^2^ > 2σ(*F*^2^)] = 0.026	*w* = 1/[σ^2^(*F*_o_^2^) + (0.0453*P*)^2^] where *P* = (*F*_o_^2^ + 2*F*_c_^2^)/3
*wR*(*F*^2^) = 0.063	(Δ/σ)_max_ = 0.001
*S* = 1.00	Δρ_max_ = 0.36 e Å^−^^3^
3310 reflections	Δρ_min_ = −0.20 e Å^−^^3^
92 parameters	Extinction correction: none
1 restraint	Absolute structure: Flack (1983), 1261 Friedel pairs
Primary atom site location: structure-invariant direct methods	Flack parameter: −0.01 (3)
Secondary atom site location: difference Fourier map	

### Special details

**Table:**

Geometry. All e.s.d.'s (except the e.s.d. in the dihedral angle between two l.s. planes) are estimated using the full covariance matrix. The cell e.s.d.'s are taken into account individually in the estimation of e.s.d.'s in distances, angles and torsion angles; correlations between e.s.d.'s in cell parameters are only used when they are defined by crystal symmetry. An approximate (isotropic) treatment of cell e.s.d.'s is used for estimating e.s.d.'s involving l.s. planes.
Refinement. Refinement of *F*^2^ against ALL reflections. The weighted *R*-factor *wR* and goodness of fit *S* are based on *F*^2^, conventional *R*-factors *R* are based on *F*, with *F* set to zero for negative *F*^2^. The threshold expression of *F*^2^ > σ(*F*^2^) is used only for calculating *R*-factors(gt) *etc*. and is not relevant to the choice of reflections for refinement. *R*-factors based on *F*^2^ are statistically about twice as large as those based on *F*, and *R*- factors based on ALL data will be even larger.

### Fractional atomic coordinates and isotropic or equivalent isotropic displacement parameters (Å^2^)

**Table:**

	*x*	*y*	*z*	*U*_iso_*/*U*_eq_	
Cl	0.85575 (3)	0.53610 (3)	0.500252 (12)	0.01541 (5)	
O1	0.08282 (10)	0.92929 (11)	0.46762 (4)	0.01799 (13)	
H11	0.0253	0.8180	0.4732	0.027*	
N1	0.56591 (11)	0.89016 (11)	0.51704 (4)	0.01256 (13)	
H1	0.6593	0.7862	0.5167	0.015*	
C2	0.36697 (13)	0.80978 (13)	0.54256 (5)	0.01451 (15)	
H21	0.3745	0.7744	0.5955	0.017*	
H22	0.3327	0.6869	0.5145	0.017*	
C3	0.20359 (12)	0.97096 (13)	0.53048 (5)	0.01341 (14)	
H3	0.1163	0.9797	0.5752	0.016*	
C4	0.30861 (12)	1.17237 (13)	0.51807 (5)	0.01337 (14)	
H4	0.2084	1.2843	0.5185	0.016*	
C5	0.46336 (13)	1.20182 (13)	0.57991 (5)	0.01436 (15)	
H52	0.3990	1.1795	0.6284	0.017*	
H51	0.5161	1.3409	0.5787	0.017*	
C6	0.63647 (13)	1.05125 (14)	0.56902 (5)	0.01397 (15)	
H61	0.7553	1.1206	0.5483	0.017*	
H62	0.6748	0.9912	0.6169	0.017*	
C7	0.54836 (14)	0.97482 (14)	0.44064 (5)	0.01571 (16)	
H71	0.4843	0.8753	0.4076	0.019*	
H72	0.6835	1.0064	0.4210	0.019*	
C8	0.42035 (14)	1.16677 (14)	0.44410 (5)	0.01546 (15)	
H82	0.5077	1.2865	0.4394	0.019*	
H81	0.3226	1.1680	0.4030	0.019*	

### Atomic displacement parameters (Å^2^)

**Table:**

	*U*^11^	*U*^22^	*U*^33^	*U*^12^	*U*^13^	*U*^23^
Cl	0.01498 (9)	0.01326 (9)	0.01800 (9)	0.00201 (7)	0.00182 (7)	0.00096 (7)
O1	0.0151 (3)	0.0194 (3)	0.0195 (3)	−0.0034 (2)	−0.0051 (2)	−0.0014 (2)
N1	0.0121 (3)	0.0123 (3)	0.0133 (3)	0.0018 (2)	0.0001 (2)	0.0000 (2)
C2	0.0139 (3)	0.0126 (3)	0.0171 (4)	−0.0022 (3)	−0.0003 (3)	0.0018 (3)
C3	0.0103 (3)	0.0151 (3)	0.0149 (3)	−0.0010 (3)	−0.0001 (3)	−0.0015 (3)
C4	0.0122 (3)	0.0112 (3)	0.0167 (4)	0.0016 (2)	−0.0018 (3)	−0.0008 (3)
C5	0.0136 (3)	0.0137 (3)	0.0157 (4)	0.0002 (3)	−0.0010 (3)	−0.0024 (3)
C6	0.0125 (3)	0.0148 (3)	0.0146 (4)	−0.0001 (3)	−0.0027 (3)	−0.0011 (3)
C7	0.0165 (4)	0.0195 (4)	0.0111 (3)	0.0023 (3)	0.0017 (3)	0.0007 (3)
C8	0.0158 (4)	0.0163 (4)	0.0143 (4)	0.0005 (3)	−0.0013 (3)	0.0034 (3)

### Geometric parameters (Å, °)

**Table:**

O1—C3	1.4227 (11)	C4—C5	1.5355 (13)
O1—H11	0.8400	C4—H4	1.0000
N1—C7	1.5009 (13)	C5—C6	1.5396 (13)
N1—C2	1.5011 (12)	C5—H52	0.9900
N1—C6	1.5031 (12)	C5—H51	0.9900
N1—H1	0.9300	C6—H61	0.9900
C2—C3	1.5430 (13)	C6—H62	0.9900
C2—H21	0.9900	C7—C8	1.5367 (14)
C2—H22	0.9900	C7—H71	0.9900
C3—C4	1.5284 (13)	C7—H72	0.9900
C3—H3	1.0000	C8—H82	0.9900
C4—C8	1.5349 (14)	C8—H81	0.9900
			
C3—O1—H11	109.5	C4—C5—C6	108.97 (7)
C7—N1—C2	110.49 (7)	C4—C5—H52	109.9
C7—N1—C6	109.64 (7)	C6—C5—H52	109.9
C2—N1—C6	109.64 (7)	C4—C5—H51	109.9
C7—N1—H1	109.0	C6—C5—H51	109.9
C2—N1—H1	109.0	H52—C5—H51	108.3
C6—N1—H1	109.0	N1—C6—C5	108.12 (6)
N1—C2—C3	109.27 (7)	N1—C6—H61	110.1
N1—C2—H21	109.8	C5—C6—H61	110.1
C3—C2—H21	109.8	N1—C6—H62	110.1
N1—C2—H22	109.8	C5—C6—H62	110.1
C3—C2—H22	109.8	H61—C6—H62	108.4
H21—C2—H22	108.3	N1—C7—C8	108.51 (7)
O1—C3—C4	108.13 (7)	N1—C7—H71	110.0
O1—C3—C2	112.11 (7)	C8—C7—H71	110.0
C4—C3—C2	107.96 (7)	N1—C7—H72	110.0
O1—C3—H3	109.5	C8—C7—H72	110.0
C4—C3—H3	109.5	H71—C7—H72	108.4
C2—C3—H3	109.5	C4—C8—C7	108.93 (7)
C3—C4—C8	109.21 (7)	C4—C8—H82	109.9
C3—C4—C5	108.12 (7)	C7—C8—H82	109.9
C8—C4—C5	108.49 (8)	C4—C8—H81	109.9
C3—C4—H4	110.3	C7—C8—H81	109.9
C8—C4—H4	110.3	H82—C8—H81	108.3
C5—C4—H4	110.3		
			
C7—N1—C2—C3	−50.50 (9)	C8—C4—C5—C6	−48.56 (9)
C6—N1—C2—C3	70.45 (9)	C7—N1—C6—C5	71.20 (8)
N1—C2—C3—O1	102.96 (9)	C2—N1—C6—C5	−50.27 (9)
N1—C2—C3—C4	−16.03 (9)	C4—C5—C6—N1	−16.88 (9)
O1—C3—C4—C8	−53.44 (9)	C2—N1—C7—C8	69.13 (9)
C2—C3—C4—C8	68.06 (9)	C6—N1—C7—C8	−51.82 (9)
O1—C3—C4—C5	−171.30 (7)	C3—C4—C8—C7	−49.82 (9)
C2—C3—C4—C5	−49.80 (9)	C5—C4—C8—C7	67.81 (9)
C3—C4—C5—C6	69.77 (9)	N1—C7—C8—C4	−15.62 (10)

### Hydrogen-bond geometry (Å, °)

**Table:**

*D*—H···*A*	*D*—H	H···*A*	*D*···*A*	*D*—H···*A*
N1—H1···Cl	0.93	2.14	3.060 (2)	171
O1—H11···Cl^i^	0.84	2.24	3.079 (2)	173

Symmetry codes: (i) *x*−1, *y*, *z*.
